# A forensic identification case and DPid - can it be a useful tool?

**DOI:** 10.1590/1678-7757-2016-0175

**Published:** 2017

**Authors:** Cristhiane Leão de QUEIROZ, Ellen Marie BOSTOCK, Carlos Ferreira SANTOS, Marco Aurélio GUIMARÃES, Ricardo Henrique Alves da SILVA

**Affiliations:** 1 Universidade de São Paulo, Faculdade de Odontologia de Ribeirão Preto, Departamento de Estomatologia, Saúde Coletiva e Odontologia Legal, Ribeirão Preto, SP, Brasil.; 2 Reynolds Community College, Richmond, VA, USA.; 3 Universidade de São Paulo, Faculdade de Odontologia de Bauru, Departamento de Ciências Biológicas, Bauru, SP, Brasil.; 4 Universidade de São Paulo, Faculdade de Medicina de Ribeirão Preto, Departamento de Patologia e Medicina Legal, Ribeirão Preto, SP, Brasil.

**Keywords:** Forensic dentistry, Forensic anthropology, Denture identification marking, Dental prosthesis, Dentures

## Abstract

**Objective:**

The aim of this study was to show DPid as an important tool of potential application to solve cases with dental prosthesis, such as the forensic case reported, in which a skull, denture and dental records were received for analysis.

**Material and Methods:**

Human identification is still challenging in various circumstances and Dental Prosthetics Identification (DPid) stores the patient’s name and prosthesis information and provides access through an embedded code in dental prosthesis or an identification card. All of this information is digitally stored on servers accessible only by dentists, laboratory technicians and patients with their own level of secure access. DPid provides a complete single-source list of all dental prosthesis features (materials and components) under complete and secure documentation used for clinical follow-up and for human identification.

**Results and Conclusion:**

If DPid tool was present in this forensic case, it could have been solved without requirement of DNA exam, which confirmed the dental comparison of antemortem and postmortem records, and concluded the case as a positive identification.

## Introduction

Crime rate, terrorism, wars, mass disasters, road traffic accidents and dreadful diseases have increased^2^. Moreover, natural or man-made disasters present a different set of circumstances and, consequently, each event results in new challenges for identification teams^18^. The identity of the deceased, assailant, or the cause of death becomes important as the core of various investigations^2^.

Resilience of the dental structures to *postmortem* assault, denture labeling, and teeth as a source of DNA contribute to make identification successful. Dental identification is widely used, not only in the single fatality situation, but also in mass fatality incidents and cases of missing persons^9^ ^,^ ^22^.

New technologies have been developed to make it faster and more effective and different disaster victim identification (DVI) protocols have been evaluated and improved. The *postmortem* (PM) and *antemortem* (AM) data are entered into a computer database that will ultimately search for best possible matches^18^.

Denture may demonstrate certain features that can assist in the identification such as old or recent repairs, areas of relief, soft linings, material used, or a particular tooth type and arrangement. If a denture wearer was involved in an accident, crime, or mass disaster, it would be invaluable if the denture was labeled. The marker should ideally withstand most conditions, be acceptable to the patient, not weaken the denture, and be easy and cheap to produce and give a positive identification^20^.

The aim of this study was to show DPid as an important tool of potential application to solve cases with dental prosthesis, such as the forensic case in which skull, denture, and dental records were received for analysis.

## Case Report

A skull, complete bones, and a denture were found in a crime scene and they were referred to the Center of Legal Medicine of Ribeirão Preto, University of São Paulo, for anthropological and dental analysis at the Laboratory of Forensic Anthropology. The skull and complete bones presented the following anthropological information: male, interbred (white + black), predominantly black appearance, age range between 38 and 57 years (average 48 years), height between 1.68 m and 1.80 m. Skull and denture were sent to maxilla and mandible exams, dental prosthesis exam, radiographs, charts, photos, *antemortem* information analysis as well as *antemortem* and *postmortem* information comparison.

**Figure 1:**
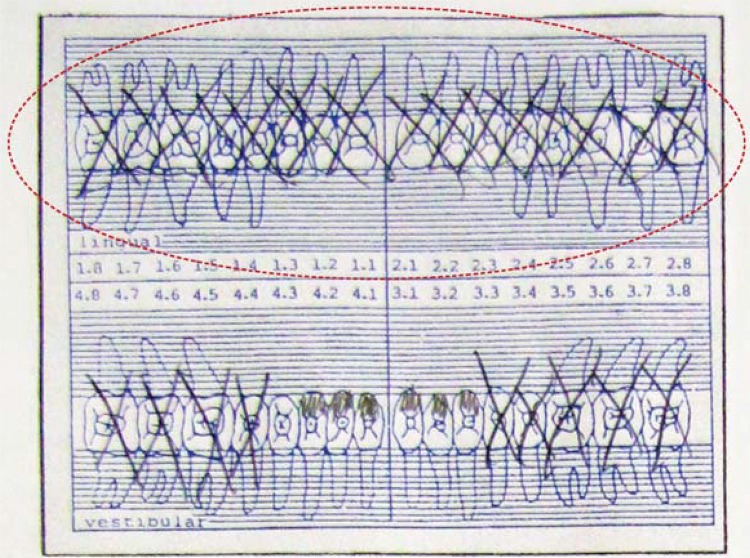
Odontogram of the suspect. Completely edentulous maxilla in initial situation. “X” means absent teeth (antemortem)

**Figure 2:**
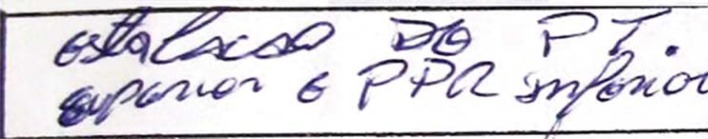
Dental treatment records. Upper denture insertion and lower partial removable prosthesis (antemortem)

**Figure 3:**
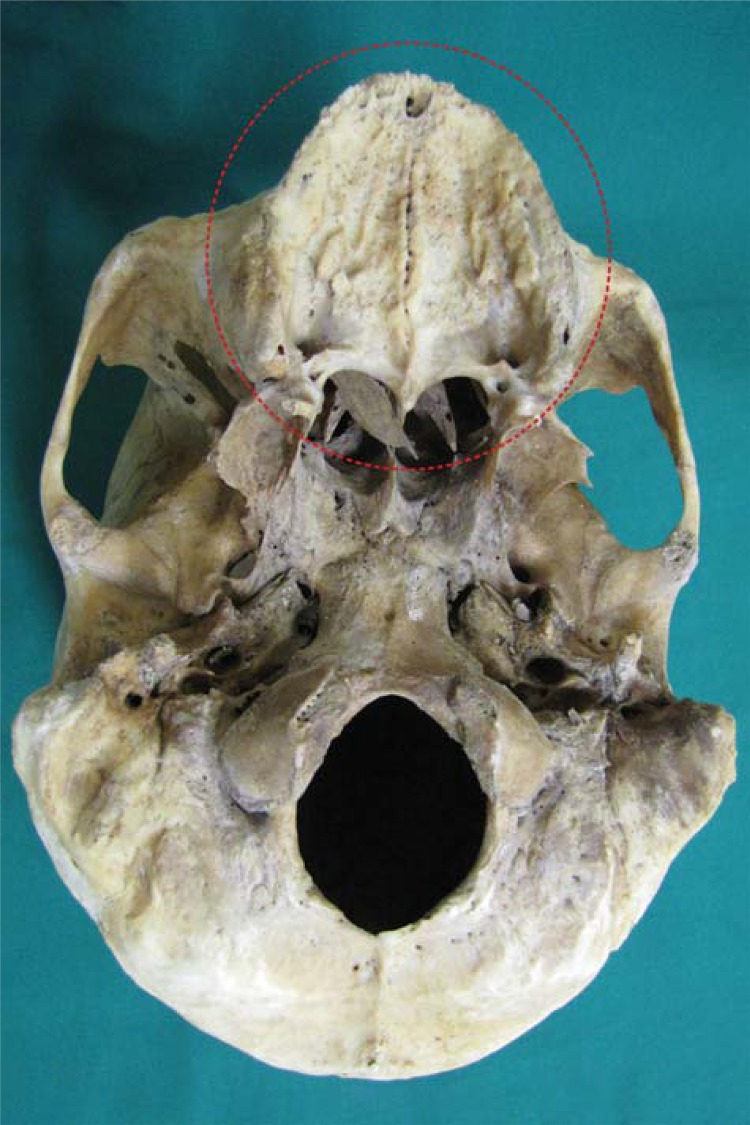
Skull referred to the Center of Legal Medicine of Ribeirão Preto, University of São Paulo, for anthropological and forensic dentistry analysis at the Laboratory of Forensic Anthropology. Completely edentulous maxilla is highlighted (postmortem)

**Figure 4:**
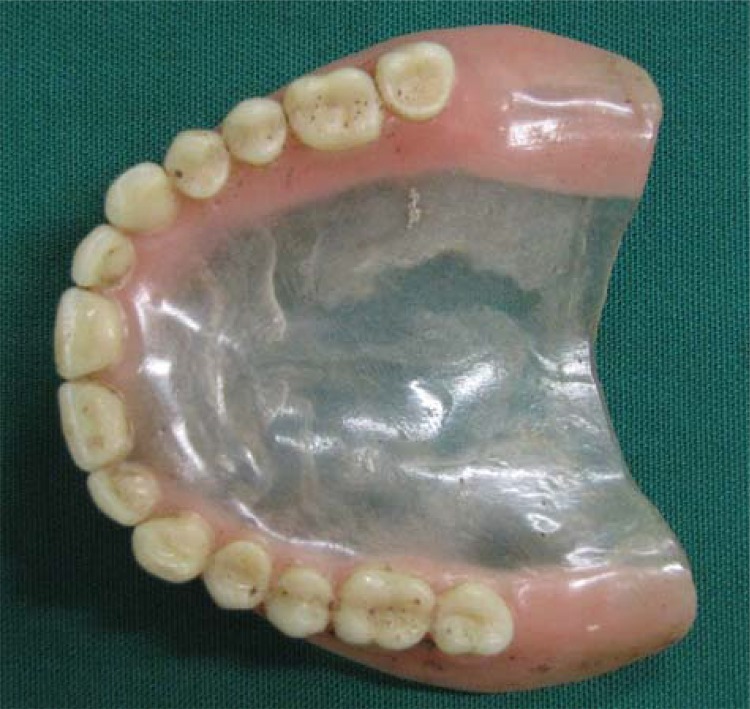
Upper denture referred to the Center of Legal Medicine of Ribeirão Preto, University of São Paulo, for anthropological and forensic dentistry analysis at the Laboratory of Forensic Anthropology (postmortem)

**Figure 5:**
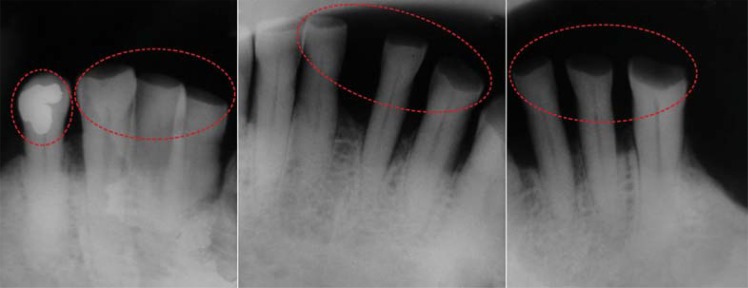
Amalgam restoration on the lower right first premolar and dental attrition on the incisal borders of the lower canines as well as central and lateral incisors (antemortem)

**Figure 6:**
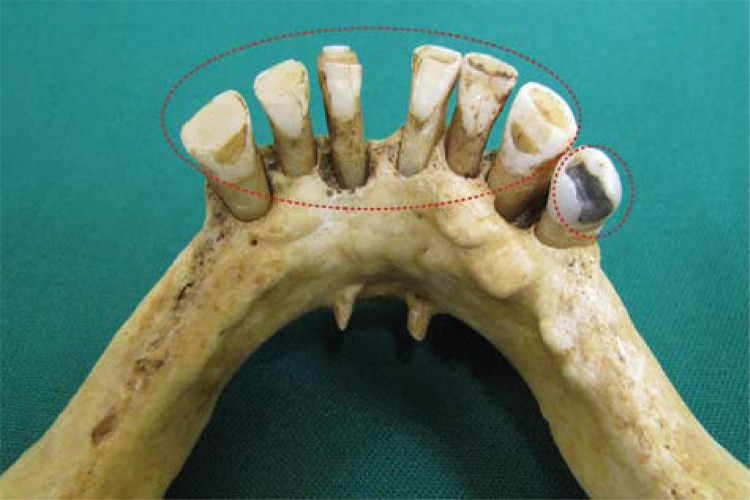
Amalgam restoration on the lower right first premolar and dental attrition on the incisal borders of the lower canines as well as central and lateral incisors (postmortem)

In intraoral clinical examination, absence of any dental element in maxilla and upper denture presence were observed. Canines, central and lateral incisors, and the right first premolar were present in the mandible. Additionally, dental records and three periapical radiographs from the suspect’s family were received. Coincidence was found between points in dental records produced *antemortem* and the *postmortem* analysis (Figures 1, 2, 3, 4, 5 and 6).

Therefore, identification from *antemortem* and *postmortem* dental records with consistent characteristics was defined, if DPid tool was present in this forensic case, it could have been solved without a requirement of DNA exam by the law enforcement agencies, which confirmed the dental comparison and concluded the case as a positive identification.

## Discussion

Identification methods must be scientifically sound, reliable, applicable under field conditions, and capable of being implemented within a reasonable period of time. The primary and most reliable means of identification are fingerprint analysis, comparative dental analysis, and DNA analysis^10^.

The positive identification of skeletal remains by individual dental parameters is one of the objectives of criminal investigation. Intervention of Forensic Dentistry in some circumstances may represent the only way to obtain a positive identification of unidentified bodies.

Human identification in Forensic Dentistry is performed through comparative and reconstructive analysis. Identification allows determining several parameters of forensic interest: specimen, population affinity, sex, age, height, and individualization factors. Forensic Dentistry is one of the most important fields in human identification, because teeth have less variability in the chronology of events in terms of the reconstructive way. On the other side, in comparative terms, this area is also important, because of the individualization factors: positive identification in individual cases and in mass disasters^17^.

Body Identification guidelines establishes some criteria such as collection and preservation of *postmortem* dental evidence: the remains – examination procedures; photography; the *postmortem* dental record: dental examination; narrative description and nomenclature; dental impressions; dental radiology; comparison of *antemortem* and *postmortem* evidence – dental features useful in identification; categories and terminology for body identification – positive identification; possible identification; insufficient evidence; exclusion^1^. These criteria were followed in the present case, in which dental positive identification was achieved before DNA exam.

Marking or labeling of dental prosthesis, primarily of dentures, for ownership identification has been occurring for years. Although there are several denture marking methods (engraving, scribing, writing, and inclusion)^4^ ^,^ ^15^, the most challenging criterion is the ability to survive a common range of chemical and thermal insults with the exception of fire^21^. In addition, these methods include variations of criteria; for instance, but not limited to, the prosthesis should not be weakened by the marking method and should be cosmetically acceptable. However, with increased globalization, and especially the patients’ ability to relocate around the world, two key elements should also be considered: security and technology.

The most commonly used form of ownership identification is inclusion or embedding the patient’s name or other personal identifier into the prosthesis. This is the easiest and least expensive approach; however, it provides no level of personal security or background information. For example, in the United States of America, ownership identification is typically done by embedding the patient’s name and/or Social Security Number (SSN) into the prosthesis^7^. A SSN is a 9-digit number assigned to each citizen with the original intent to monitor work history, but it has since become a primary identification source. Displaying the patient’s name and/or SSN poses a considerable personal security risk because one in every 10 American consumers^12^ and approximately 11% of people over the age of 65 years have their identity stolen^19^.

In recent years, techniques using newer technologies with a higher level of security have been introduced such as microdots, Radio Frequency Transponder (RFID) chips^14^ ^,^ ^16^, and memory card^11^. Unfortunately, the cost associated with these technologies, both in the item to be embedded and the specialized reader/scanner necessary to extract the information, prohibits them from being broadly accepted by dental professionals.

**Figure 7:**
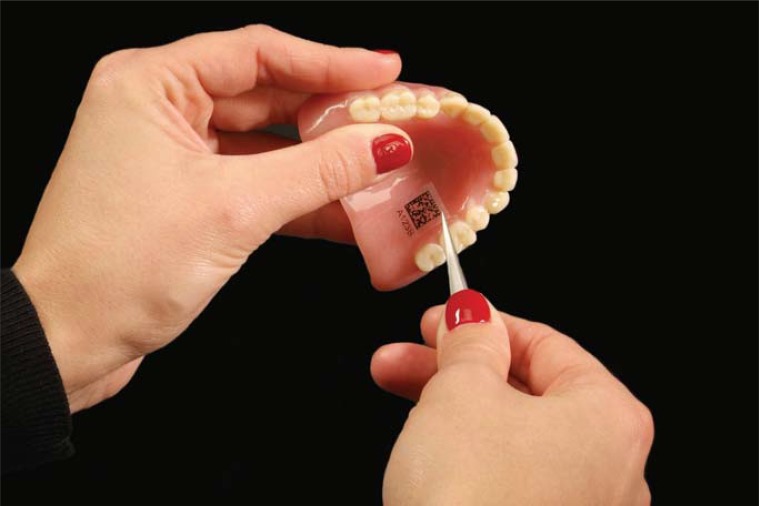
Embedded DPid code in a denture^6^

**Figure 8:**
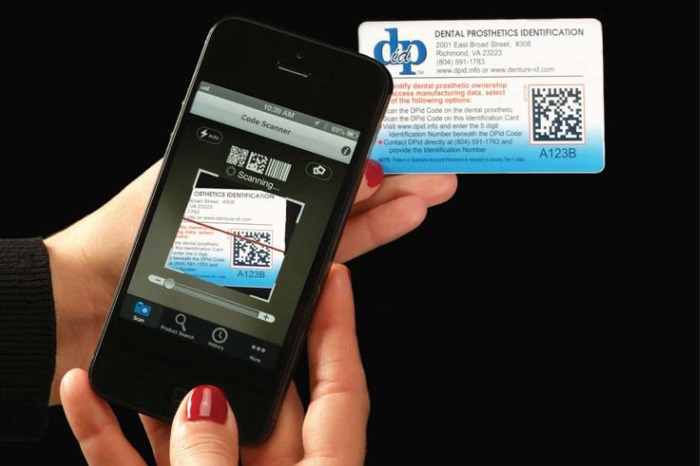
DPid code scanned using a smartphone in an identification card^6^

Dental Prosthetics Identification (DPid) provides a viable alternative/solution to ownership identification using readily available and inexpensive technology in addition to providing a high level of personal security. The DPid system uses both an embedded code (Figure 7)^6^ and an identification card (displaying the same code) (Figure 8)^6^ providing an alternative means of accessing patient identification and prosthetic information. The identification card may also appeal to patients who wish not to have a code embedded in their prosthesis or if their prosthesis is not physically capable of having a code embedded such as a crown or a bridge. Also, because the code and 5-digit alpha/numeric number are associated, they are interchangeable. Then, if the prosthesis is physically too small to accept a code or the patient is not interested in having a code embedded into the prosthesis, the smaller less obtrusive identification number may be used in its place.

**Figure 9:**
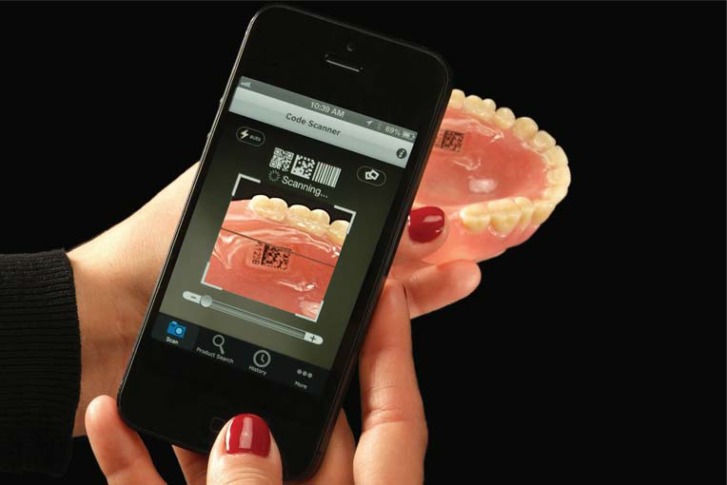
DPid code scanned using a smartphone in a denture^6^

DPid system randomly generates a unique 2D Data Matrix Code associated with a 5-digit alpha/numeric number for each patient. DPid code may be scanned using either a smartphone (Figures 8 and 9)^6^ or a tablet equipped with a 2D Code Reader App (typically free) or by logging onto the DPid website and entering the patient’s 5-digit alpha/numeric number.

Access to a patient’s identification and prosthetic information can only be gained by entering a password. However, if a patient is a resident at a nursing home or similar care facility, the password requirement may be omitted with the patient’s consent. All patient data are encrypted and stored on secure dedicated servers and are available only to dentists, laboratory technicians, and patients, each with their own level of secure access.

DPid system has two secure levels with Tier 1 including: the patient’s first, middle, and last names; all essential data listed on the Dental Prosthetic Categories to manufacture, repair, or remanufacture of the dental prosthesis including photographs and case notes; specific listing of U.S. Food and Drug Administration Class II Dental Medical Device codes and numbers^5^ ^,^ ^23^; manufacture date; manufacturing point-of-origin; dental laboratory business name, address, phone, e-mail, contact person; if the dental laboratory is a Certified Dental Laboratory Technician (CDT) on staff, Certified Dental Laboratory (CDL), Dental Appliance Manufacturer’s Audit System Certified Laboratory (DAMAS), or a U.S. State-Registered Dental Laboratory; dentist business name, address, phone, e-mail, dentist name, and dentist license number; notification of pertinent health information (Tier 2) about the patient’s dental well-being regarding the manufacturing materials or the procedure of inserting the dental prosthetic.

All dental prosthesis, removable or fixed, can be identified with DPid system. Therefore, if a patient needs to have the prosthesis repaired or remanufactured, the laboratory technician can rapidly access all the necessary information via the embedded code or identification card. All information is entered in “real-time”, in such a way the dentist and laboratory technician can access the patient’s information and see the exact same data including materials and components, photographs, and case notes. If the dentist uploads photographs of the patient’s existing condition, the laboratory technician can see the condition of the patient’s existing dentition and what the dentist is proposing as a solution.

Dental professionals using the DPid system can be trained on how to enter and access data. For others who do not need this information, the DPid code will, in most cases, remain undetectable, as intended for the patient’s aesthetics and privacy. However, First Responders (Fire and/or Emergency Medical Services) along with Forensics Experts who arrive on the scene to a nonresponsive or deceased person will follow specific processes and procedures in determining the identity of the person, which may include accessing removable dental prosthetics. Under these circumstances, First Responders are typically trained and equipped with smartphones or electronic tablets to log critical events, vitals, medications, actions, and logs. Because of this training, a First Responder should recognize the DPid code as a scan image and have a basic understanding of how it works. When contacted, DPid has processes and procedures in place to assist First Responders in emergency situations.

Regarding Forensic Dentistry, timely access to patient identification and current dental records can be crucial. Thus, marked dental prostheses (full and partial dentures, mouth guards, and removal orthodontic appliances) would lead to rapid identification in the event of accidents and disasters^8^. Having access to the patient’s dental prosthetic history and progression of prosthetic applications, such as crown, bridge, denture, attachment with implants, would further confirm identification, in addition to supporting photographs and case notes. The ability to have access to this information from anywhere around the world via the internet or mobile telecommunications technology would reduce valuable time and assets in the investigation process.

There are further complications with the purging of patient dental records. Archiving of patient information varies between countries, considering that the U.S.A. typically maintains records for six years^3^, while in Brazil the dental records should be archived throughout the patient’s life^13^. Unfortunately, once the patient’s file is purged, there is no longer a record that can link the prosthesis to a patient. DPid digitally and securely stores dental prosthesis information archived and makes it accessible for patient’s lifetime.

## Conclusions

Dental comparative analysis is one of the main human identification methods through individual dental parameters that help in a positive identification of unidentified bodies. Forensic cases with dental prosthesis presence can easily achieve positive identification if there is an identification system, embedded in the prosthesis. So, in situations as the case presented, DPid is a tool that can reduce information management time, eliminate paperwork, and digitally archive patient’s information with no need for expensive programing, technical support or equipment and it will immediately reduce or eliminate expenses for research time, storage, printer ink and paper, as well as information transfer between dentists and laboratory technicians and patients when material disclosure is requested. This type of system offers a complete open-line of communication between the patient, dentist, and laboratory technician concerning documenting, identifying, and tracking all dental prostheses. Therefore, it can help to solve forensic cases, especially identification cases.
